# A water cooled, high power, dielectric barrier discharge reactor for CO_2_ plasma dissociation and valorization studies

**DOI:** 10.1038/s41598-023-33241-9

**Published:** 2023-05-06

**Authors:** Nicola Lisi, Umberto Pasqual Laverdura, Rosa Chierchia, Igor Luisetto, Stefano Stendardo

**Affiliations:** ENEA Casaccia, Via Anguillarese 301, 00123 Rome, Italy

**Keywords:** Plasma physics, Chemical engineering, Electrical and electronic engineering, Energy, Environmental chemistry, Green chemistry, Physical chemistry, Environmental sciences, Energy science and technology

## Abstract

Aiming at the energy efficient use and valorization of carbon dioxide in the framework of decarbonization studies and hydrogen research, a novel dielectric barrier discharge (DBD) reactor has been designed, constructed and developed. This test rig with water cooled electrodes is capable of a plasma power tunable in a wide range from 20W to 2 kW per unit. The reactor was designed to be ready for catalysts and membrane integration aiming at a broad range plasma conditions and processes, including low to moderate high pressures (0.05–2 bar). In this paper, preliminary studies on the highly endothermic dissociation of CO_2_, into O_2_ and CO, in a pure, inert, and noble gas mixture flow are presented. These initial experiments were performed in a geometry with a 3 mm plasma gap in a chamber volume of 40cm^3^, where the process pressure was varied from few 200 mbar to 1 bar, using pure CO_2_, and diluted in N_2_. Initial results confirmed the well-known trade-off between conversion rate (up to 60%) and energy efficiency (up to 35%) into the dissociation products, as measured downstream of the reactor system. Improving conversion rate, energy efficiency and the trade-off curve can be further accomplished by tuning the plasma operating parameters (e.g. the gas flow and system geometry). It was found that the combination of a high-power, water-cooled plasma reactor, together with electronic and waveform diagnostic, optical emission and mass spectroscopies provides a convenient experimental framework for studies on the chemical storage of fast electric power transients and surges.

## Introduction

The large scale and energy efficient handling of gases relevant for energy cycles, both related to human activities and natural processes, from volcanic to natural-biological, is an historical goal for human technology while it poses several scientific, multi-disciplinary challenges. Indeed, the chemical gas phase transformations between H_2_, H_2_O, O_2_, CO_2_, CO, N_2_, NH_3_, CH_4_ and higher hydrocarbons accounts for the greatest part of the energy exchange of natural and human related processes on earth surface and for greenhouse gases emission into the atmosphere.

Beyond the technological feasibility of interfering with such grand scale planetary system apart from the ubiquitous oxidation combustion, gaining the practical knowledge from the basic science to the technological details on energy storage and transformation is a mandatory premise to any “ecological transition” that does not imply a drastic reduction of human (lives and) wellness on earth.

The concept of using CO_2_ plasma dissociation to implement large scale energy storage was developed in the late 70 s mainly by Legasov’s group^1^. At the time the issue was the abundant availability of nuclear energy during night hours, and it was proposed that hydrogen could be produced by CO_2_ plasma dissociation, CO/O_2_ separation, and the downstream reaction of CO with water into H_2_ (and CO_2_) as an alternative to water electrolysis. Due to the extremely fast response time of plasma power systems, the same concept is attractive for being applied to renewable electric energy transients and surges, to implement a closed loop “power to gas” energy storage scheme into H_2_. Further, the simultaneous presence in the same plant of H_2_ and CO suggests that “open loop” reaction pathway may become convenient based on electric renewable availability and forecasts, on grid and fuel requirements to produce electric fuels (known as e-fuels).

**Figure 1:**
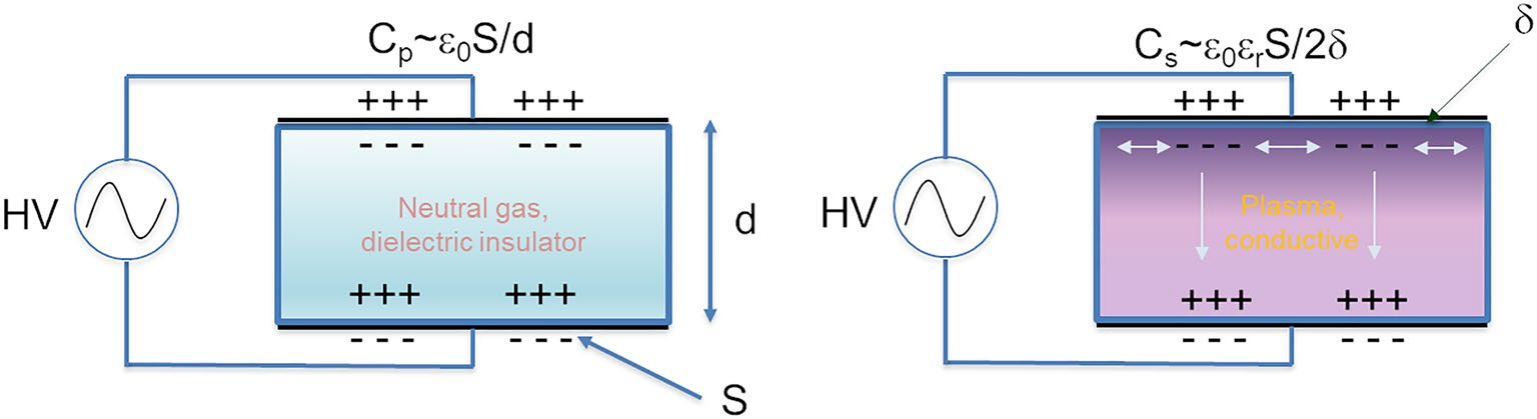
The DBD mechanism, induced charges are shown for the positive half-wave. Cp is the parasitic capacitance while Cs is the capacitance during the discharge.

Indeed, in the laboratory scale, these early studies found and reported high dissociation energy efficiencies: 80% for subsonic and 90% for supersonic flow, for optimized gas pressure, electron density and electron energy in microwave excited plasmas^2^. Conversely high frequency (HF, in the 100 kHz range) driven dielectric barrier discharge (DBD) plasmas^3^ are more interesting then microwave (MW) plasmas^4^ to practically apply to the concept due to several advantages: low cost, high electric driver efficiency (i.e. wall plug to plasma), high average power drivers with low cost components, avoidance of MW matching networks, and scale-up to industrial size (as for ozonizers^5^). Contrarily to Direct Current Glow Discharges^6^, DBD plasmas are easily stabilized at high pressure (i.e. atmospheric and above^7^) as they intrinsically prevent thermal runaways on electrode surfaces by injecting a limited charge per cycle. In Fig. 1 we show a scheme of the DBD plasma, where the gas breakdown is induced by an alternating high voltage applied across gas filled dielectric wall enclosures, as charges are capacitively induced onto the inner walls dielectric surface and move along the inner surfaces (surface discharge) and across the gap (gas discharge).

Several different plasma systems have been applied to CO_2_ dissociation studies since then, based on DBD^7–13^, glow discharges^6,14,15^, microwaves^4^, gliding and rotating arcs^11,16–20^ both for direct CO_2_ disintegration and towards its valorization via methanation^11,21,22^, dry reforming^23–25^ and for the production of liquids^26^ when catalytic systems are added to the discharge region. To our best knowledge, the highest efficiency reported to this day is up to 43%, for a glow discharge (GD) (in fact a rotating arc) system^7^ and 50% for a large microwave discharge operating at 4 kW^27^ However, none of these studies involved the use of high power (kW), water cooled DBDs.

A common feature which was reported from the early days is the trade-off between CO_2_ dissociation rate and energy efficiency^2,28^. High energy efficiency was observed only with low conversion rates, the reason being the competition of the plasma back-reaction of upstream dissociated CO and O_2_. This phenomenon sets stringent requirements on the separation efficiency of the discharge products downstream of the plasma, mainly CO from O_2_ if one has to convert CO into H_2_ by water–gas shift (WGS) reaction and suggests the integration of O_2_ selective membranes in the plasma region^29^.

**Figure 2:**
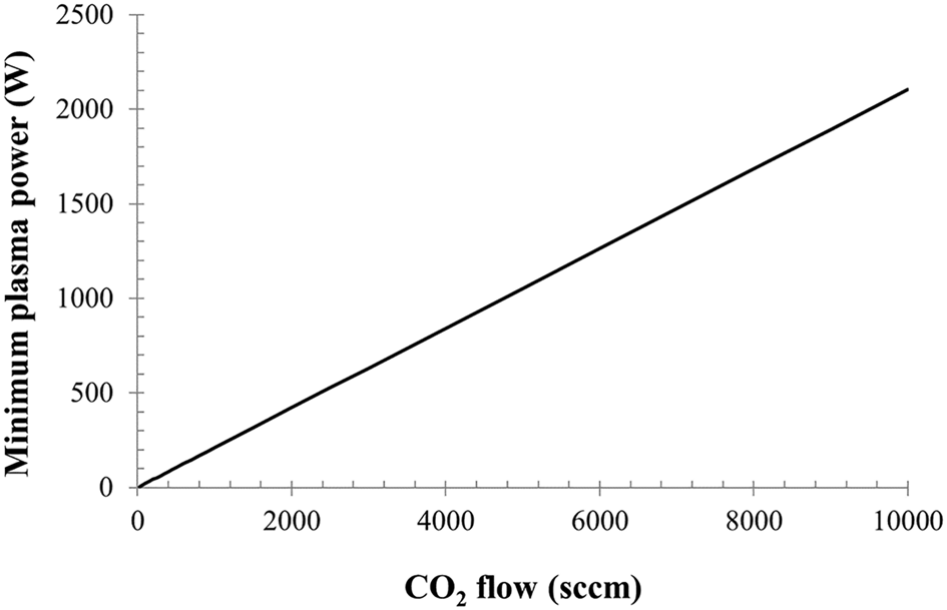
The power required to dissociate a CO_2_ flow, based on a reaction enthalpy of 283 kJ/mol.

The plasma power should generally match the dissociation enthalpy of the CO_2_ inlet flow, and it is plotted in Fig. 2, based on a reaction enthalpy of 283 kJ/mol. As a rule of thumb for the dimensioning of the system, about 1 kW is necessary to dissociate 5 Nl/min of CO_2_ assuming 100% efficiency.

In this work we are presenting the operation a DBD reactor with a power design above 1 kW, up to 2 kW to match CO_2_ flows up to 10 Nl/min. At this power water cooling is necessary to thermally stabilize the system, it also cools the electrodes surface aiming at limiting the back-reactions and improves the dielectric coupling while preventing ozone formation outside the reactor. The reactor was operated with CO_2_ was both fed as a pure gas, as from some “carbon capture” process mixed with N2, as from “combustion exhausts”, however the tested mixtures were not analytical and for reactor characterization only.

Here is a brief outline of the paper: first we introduce a scheme of the reactor, its driver circuit design, and the experimental apparatus. Then we present the characterization of the reactor and dissociation process in various operating conditions, with varying plasma power, pressure and CO_2_ flow. Finally, we further discuss in detail some scaling law for the dissociation of CO_2_ in a DBD, in view of a system optimization for efficient dissociation in high power DBDs in the light of the available literature.

## Materials and method

### Reactor system design

For this study, we developed a DBD reactor with the following characteristics: > 1 kW power, operation from vacuum to two bar absolute pressure, single quartz barrier on outer electrode, grounded inner electrode and high voltage outer electrode, water cooled inner, outer dielectric and electrode, positioned in vertical geometry.

Water cooling, a characteristic inspired by other high power DBD systems such as “excilamps”^3,5^ popular in the 90’s, has been selected for several reasons: limit CO + O_2_ recombination issues by avoiding the presence of hot metal surfaces, attain long term thermal stability during operations and similar thermal conditions under different plasma power regimes, improve the dielectric coupling (i.e. the electrical contact) between the outer steel mesh electrode and the dielectric barrier (water has ε_r_ = 80), and inhibit ozone formation in the air around the reactor. Since we are feeding running tap water for cooling through 4 mm inner diameter Teflon tubes, in our laboratory this adds a 400 kΩ resistance to ground (measured at low DC voltage). When powering the circuit, no gas bubble formation (i.e. electrolysis) was observed in the water near the outer steel mesh electrode and no increased oxidation was observed on the outer electrode.

**Figure 3:**
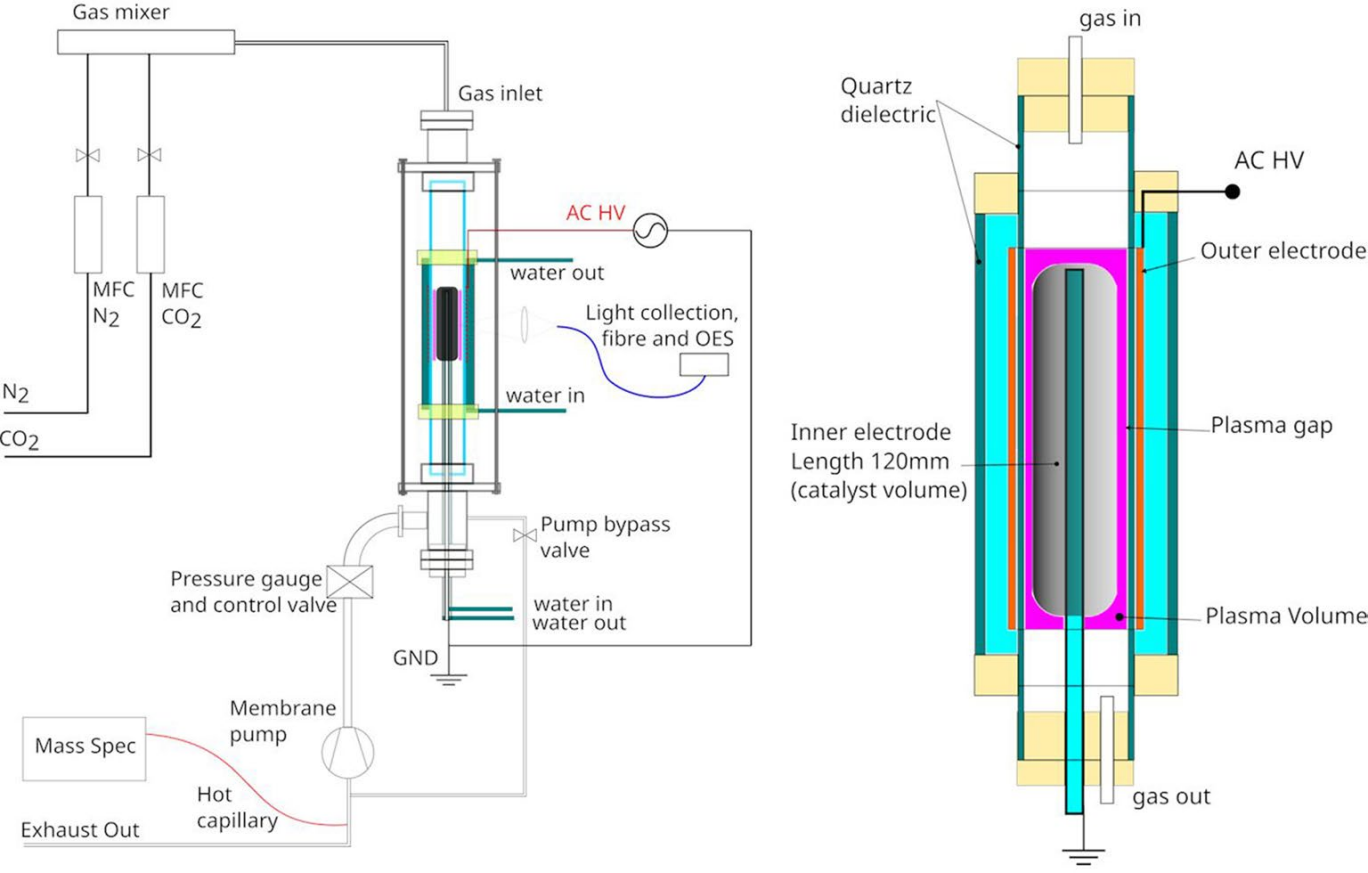
A general scheme of the experimental apparatus (left) and of the vertical, water-cooled, DBD electrode and discharge region (right).

The reactor itself is based on a 0.5 m long, 40 mm diameter quartz tube with 2 mm thickness (36 mm inner diameter) that acts as the dielectric barrier. The inner electrode assembly is based on a water cooled 10 mm diameter stainless steel support rod, that can be fitted with larger diameter coaxial electrodes to reduce the discharge gap over a selected reactor section (currently 120 mm) facing the immersed outer electrode. A general scheme of the experimental apparatus and of the electrode configuration is reported in Fig. 3.

Gases are fed by digitally controlled Mass Flow Controllers (MKS 1178) equipped with cut-off pneumatic valves, which have been calibrated by means of a bubble column. The gases are mixed and then fed from the top into the reactor, where they flow across the plasma region, and exit the reactor from the bottom.

The reactor is equipped with vacuum flange fittings and can either operate at low pressure, operating a membrane pump in conjunction with a pressure control valve (MKS 235B) and controller (MKS 600 Series) and an absolute capacitive gauge (MKS 626D), or at atmospheric pressure, when we bypass the pressure regulating set-up to a common exhaust line, where the gas is sampled. The reactor is vacuum tight, and it can maintain vacuum to the mbar level for days, therefore, since no deposition can be observed on the electrodes, the mass outflow, close to the sampling region, equals the inflow, while the molar outflow increases due to the mole increase in the reaction.

The reactor driver was built around the concept of an AC resonant discharge circuit to achieve high power and high electric efficiency. Basically, in the primary circuit two IGBT (Insulated Gate Bipolar Transistors) switch a low voltage capacitor bank (up to 400 V, charged by a Variac) at a selected frequency and voltage, which can be both manually controlled by the operator. The circuit is powering the primary winding of a custom made, ferrite core, high voltage transformer, one of the high voltage terminals of the secondary winding is directly connected to the outer electrode (immersed in cooling water and in intimate contact to the outer quartz tube) while the other to the inner grounded electrode. The transformer secondary winding and overall circuit inductance “L” are designed for an “LC” value such that the circuit becomes resonant at a target operation frequency, on the basis of the parasitic capacitance “C” of the reactor (i.e. Cp, the capacitance when the reactor system is not filled with plasma). Tuning the primary circuit switching frequency over the correct range (currently from 50 to 160 kHz) will result in large increase of the discharge voltage near resonance, that leads initially to the formation of the discharge in the gap and then to the fine tuning of the power fed to the plasma over a narrow frequency range. Namely, as the driver voltage and the power do increase in the process, and the reactor becomes more and more filled with the plasma, its circuit capacitance increases and the resonant frequency shifts to lower values, this non-linear process makes frequency tuning necessary.

**Figure 4:**
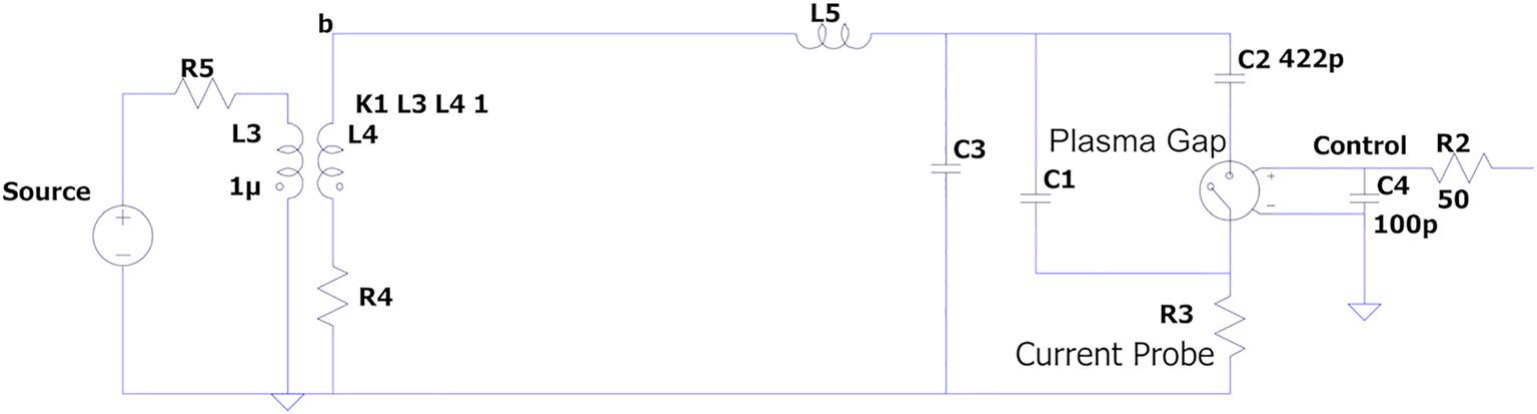
The circuit scheme of the reactor driver. Here C2 is the capacitance during discharge, while C1 and C3 are the parasitic capacitances.

A simplified scheme of the driver circuit is reported in Fig. 4, as a part of the LTSpice^30^ calculations that were performed for the initial system dimensioning, design, and development. Here the primary circuit switches are replaced by either AC or square wave sources and the (complex) plasma is simplified as a behavioral voltage source, which can have an open and closed state, with different resistances, depending on gap voltage and current. Despite its simplicity such approach allowed to dimension the circuit components, based on known CO_2_ breakdown values^31^ and on calculated capacitance values based on the physical dimensions and dielectric constants of the assembly. Here L3:L4 make the main HV transformer that steps up a pulsed current source, L5 is a parasitic inductance, C1 and C3 give the open circuit (no discharge) capacitance, while C2 is the reactor capacitance when filled with plasma. The control circuit (not shown here) can drive through R2 and C4 the pulses that switch the “plasma gap” component (a behavioral voltage source) into two different resistive states for conduction and insulation.

### Diagnostics

The main electrical discharge diagnostic and power measurement are performed by in-line voltage and current measurements: a 1Ω shunt resistor current probe bridges the inner electrode to the ground (R3 in Fig. 4), a high voltage high frequency probe (Tektronix 6025A) is in direct contact with the outer electrode, and both are fed to an oscilloscope (Tektronix TDS 220) connected to a PC by a GPIB interface. By performing a point-to-point product of the discharge current and voltage waveform with a spreadsheet software, the electrical power fed to the discharge can be calculated^32,33^, as the average of the resulting product trace over an integer number of voltage periods. An Optical Emission Spectroscopy (OES) system based on fiber coupled spectrometer (Ocean Optics HDX1100-XR) can record the light emitted by the plasma from 200 nm to 1.1 μm, which is then analyzed with specific software (Specline, by Plasus Software) for atomic and ionic transitions.

Gaseous products are measured with a Mass Spectrometer (Hyden Analytical HPR-20 R&D Gas Analysis System, equipped with corrosive gas kit) which samples the gases downstream of the reactor, the time traces of dissociated products are put in relation to the acquisition time stamp of the other diagnostics to build efficiency curves. The gas is sampled using a heated quartz capillary downstream the membrane pump during the low pressure (below atmospheric) experiments and the bypass at atmospheric pressure. The concentration of the main gas CO_2_, CO, O_2_, N_2_ is detected and registered continuously with the QGA 2.0.4 software of Hyden Analytical as volumetric percentages. Calibration is carried out with multi-points calibration method using different calibration mixtures of the gases, the detector used for this analysis is a Faraday cup, the electron energy is set at 70 eV and Emission current set at 400 (uA). The calibration is carried out at optimum vacuum in the mass spectrometer at 1.97 10^–7^ mbar adjusted also during the tests at this value. The flow during the calibration was set at 400 sccm/min, after the calibration mixtures with different flows were tested to evaluate the precision of the calibration. For the characterization of the possible minor compounds (NO_x_) formed during the tests with nitrogen is used the Hyden Analytical software MASsoft to check the ppms produced, with the same instrument parameters. Analysis and calibration are performed for different mass fragment in the case of test with and without nitrogen, in the first case CO_2_ was measured at mass 44, in the second CO_2_ mass was measured at mass 45 in this case the mass of nitrogen is measured at 29 in order to separate the two. An ABB gas analyzer with a URAS 16 modules on the exhaust gas was also applied in a test run to countercheck CO_2_ and CO concentration values.

The CO_2_ concentration was measured at the beginning and during the plasma discharge; the moles of CO_2_ were calculated assuming that the only reaction was the dissociation reaction. Other reactions do occur, but the concentration of other species such as O_3_ (for pure CO_2_ dissociation only) or nitrogen oxides (for CO_2_/N_2_ mixtures) is negligible and was not detected in significant amounts by mass spectrometry measurements. Furthermore, the calculation of the conversion via the CO_2_ content in the feed and in the exhaust, gas is within experimental error, as already reported in the literature^12^. Hence the conversion was calculated as in Eq. (1):

1 $${X}_{{CO}_{2}}\left(\%\right)=\frac{{F}_{tot,in}\cdot {\gamma }_{{CO}_{2},in}-{F}_{tot,out}\cdot {\gamma }_{{CO}_{2},out}}{{F}_{tot,in}\cdot {\gamma }_{{CO}_{2},in}}\cdot 100,$$

where ${F}_{tot,in}$ is the total flow of gas in the inlet or outlet respectively, ${\gamma }_{{CO}_{2},in}$ is the volume percentage of CO_2_ at the inlet measured at the start of the test with the mass spectrometer without the plasma, ${\gamma }_{{CO}_{2},out}$ is the volume percentage of CO_2_ at the outlet when the plasma is on.

In the case of mixtures containing N_2_ the concentration of the gases studied with the mass spectrometer were the CO_2_ at mass 45, the N_2_ at mass 14, and the O_2_ at mass 32; the measure of CO was neglected since the contribute of CO on the mass 29 was deemed neglectable at concentration below 30% v/v of CO (relative intensity of 14 mass on CO equal to 3:1000 and of mass 14 on N_2_ equal to 60:1000). In this condition since were detected only ppm of NO_x_ compounds, and the main reaction is the dissociation of CO_2_, then the N_2_ was taken as constant throughout the reaction and the Eq. (2) were applied for estimating the CO_2_ conversion, and the contribution of CO_2_, N_2_ and CO are uncoupled:

2 $${X}_{{CO}_{2}}\left(\%\right)=\frac{{F}_{tot,in}\cdot {\gamma }_{{CO}_{2}\_45,in}-{F}_{tot,out}\cdot {\gamma }_{{CO}_{2}\_45,out}}{{F}_{tot,in}\cdot {\gamma }_{{CO}_{2}\_45,in}}\cdot 100,$$

where ${\gamma }_{{CO}_{2}\_45,in}$ and ${\gamma }_{{CO}_{2}\_45,out}$ are the volumetric fraction of CO_2_, measured at mass 45, in the feed and with plasma respectively.

The efficiency was therefore calculated from the power discharge in the plasma *(P*_*plasma*_*)* and the theoretical power required to completely dissociate CO_2_ *(P*_*req*_*)* (5):

3 $$\eta \left(\%\right)=\frac{{X}_{{CO}_{2}}*{F}_{{CO}_{2}}\left(Nl/min\right)}{22.414\left(Nl/mol\right)}\cdot 60\cdot \frac{{P}_{req}\left(\frac{W\cdot s}{mol}\right)}{{P}_{plasma}\left(W\right)}.$$

The carbon balance (C%) necessary to evaluate the possible deposition of carbon residues during plasma was calculated using the formula (4):

4 $$C\left(\%\right)=\frac{{\gamma }_{{CO}_{2},out}+{\gamma }_{CO,out}}{{F}_{C{O}_{2,in}}}\cdot 100.$$

## Results and discussion

The initial experiments were performed to determine the operating ranges and to tune the hardware to avoid arcing and other operational issues due to inadequate insulation and electrode sizing, that may lead to undesired discharges along insulators, cables, and the transformer core. Since we want the secondary, high voltage AC circuit to resonate across the electrode gap, care must be placed to avoid unwanted, parasitic capacitive coupling and overvoltage across insulators.

A series of experiments were then conducted varying power, pressure, and total gas flow, both in pure CO_2_ and in various ratios of N_2_/CO_2_, measuring conversion and plasma power in all cases.

**Figure 5:**
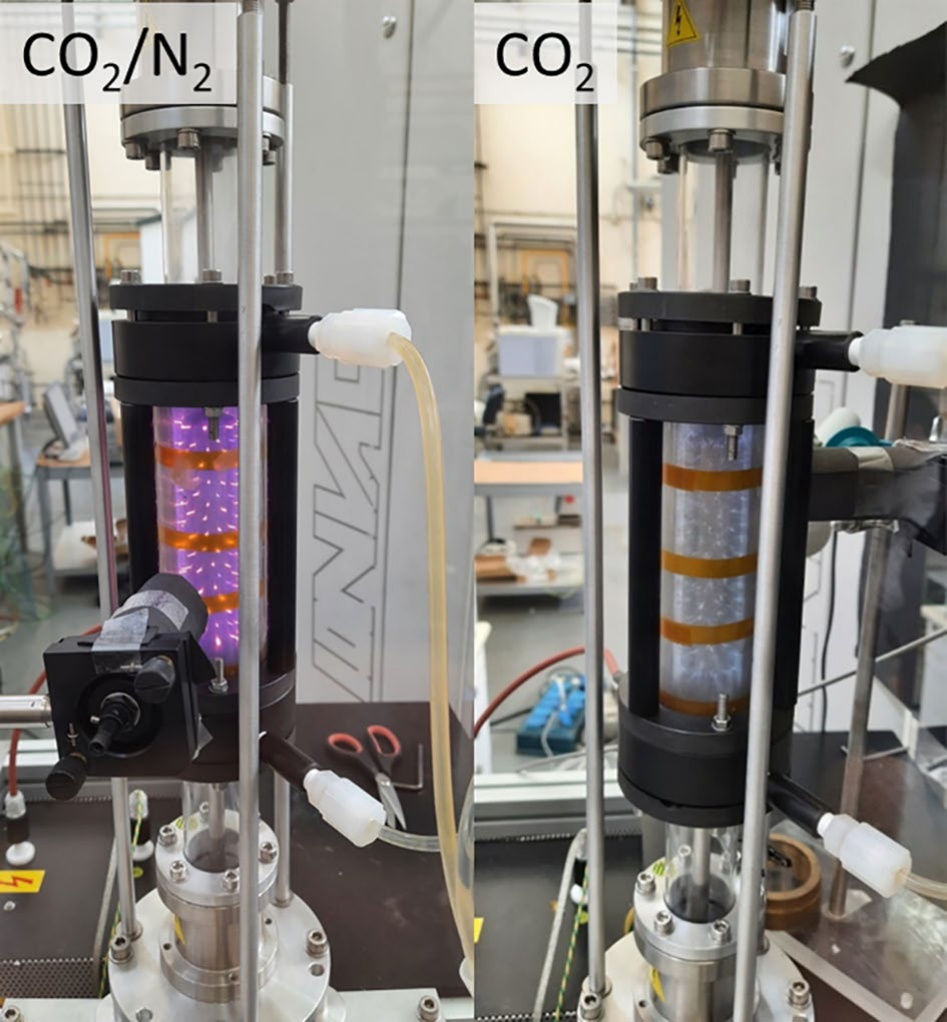
Two photographic images of the reactor during operations, on the left with CO_2_/N_2_ mixture and on the right with pure CO_2_.

In Fig. 5 we show a photographic image of the reactor during operations, on the left with CO_2_/N_2_, and on the right with a pure CO_2_. The obvious visual difference is due to the optical emission from N near 400 nm and the absence of strong visible emission lines for CO_2_.

Visually, the DBD discharge consists of several micro-discharges that move across the electrode surfaces and cover the electrode surface by slowly migrating to fill the surface and volume. The individual micro-discharges can also be seen as current spikes in the oscilloscope’s traces of the shunt probe resistor signal, each lasting 10 s of ns^5^.

Moreover, since we did not observe the formation of solid carbon on the inner electrode nor on the dielectric surface, coking could be excluded.

**Figure 6:**
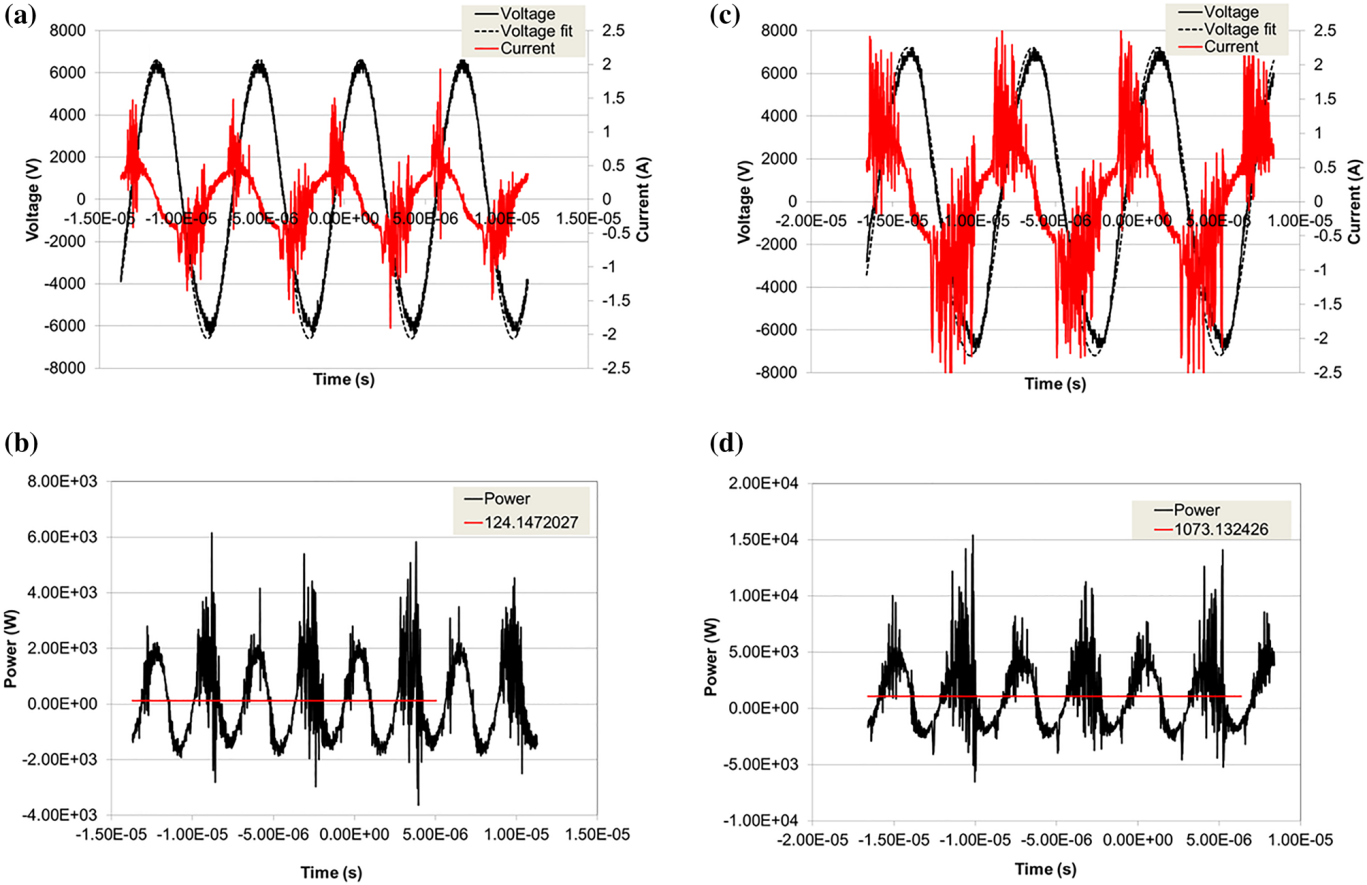
Typical voltage and current waveforms, the power waveform and average power calculation. Here on the left, (**a**) and (**b**) the discharge operating at low power, on the right (**c**) and (**d**), at high power. Pressure is 1 bar in a 1000 sccm CO_2_ 60 sccm N_2_ mixture.

In Fig. 6 we show the oscilloscope traces of two typical voltage and current waveforms as measured respectively with a high voltage probe and as a voltage drop across a shunt resistor as well as the calculated plasma power.

As an example, we show on the left pane of Fig. 6a and b the waveforms for a discharge operating at low power and on the right side, (c) and (d), at high power, at 1 bar in a 1000 sccm (standard cubic centimeter per minute) CO_2_ 60 sccm N_2_ mixture. It should be noted that care should be taken while measuring the current waveforms with the oscilloscope due to the rapid fluctuation in the waveforms, the spikes related to individual streamer’s discharges, so that either a high bandwidth probe is used, or a capacitor must be placed across the shunt resistor. However, appropriate time scale, bandwidth, and sampling rate should be selected to avoid misestimating the plasma power. We later confirmed the correctness of the measured power by measuring AC power absorption by the supply with voltage and current probes.

**Figure 7:**
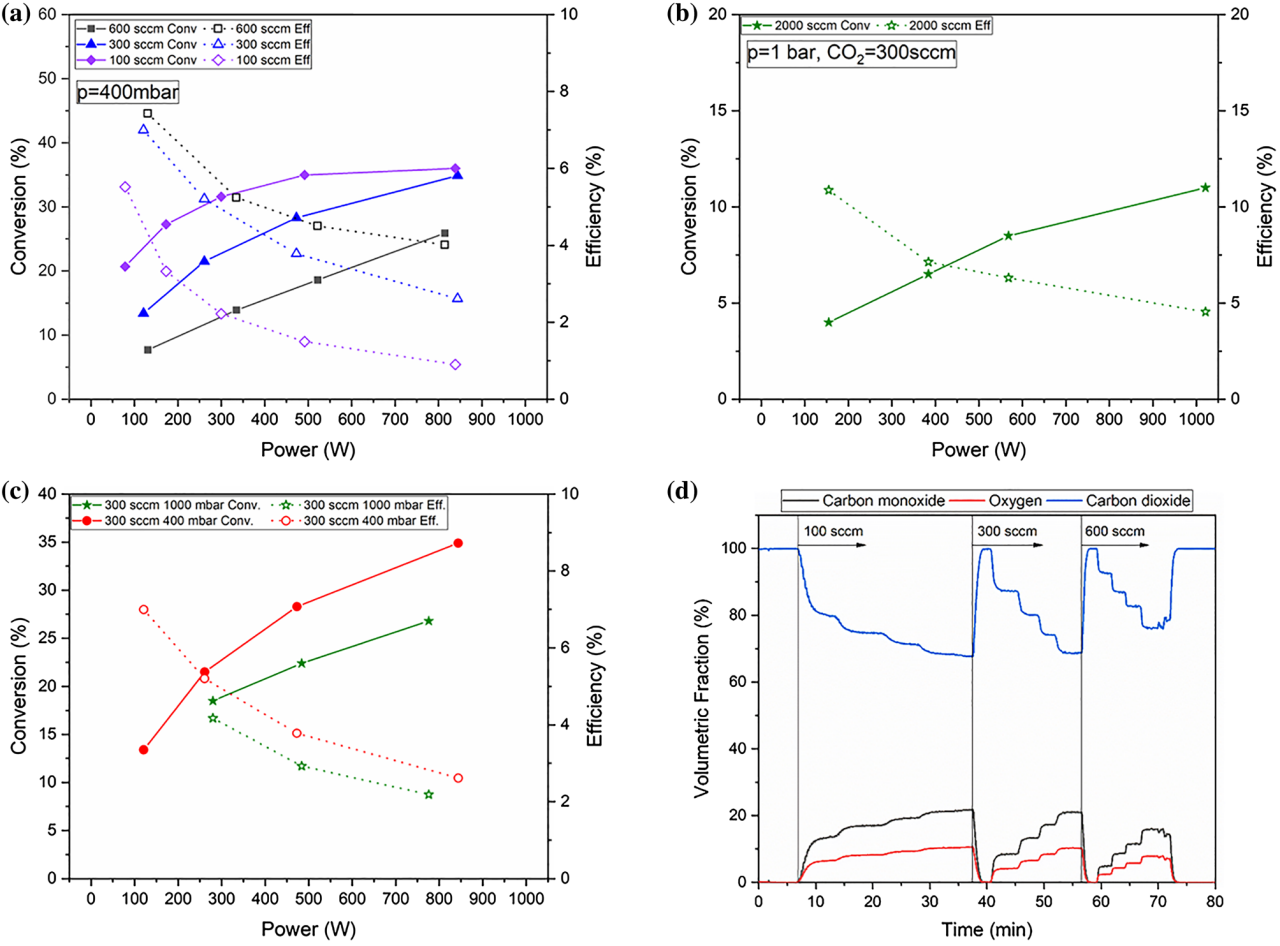
Conversions and efficiencies for pure CO_2_ flows: (**a**) pressure of 400 mBar and flow ranging from 100 to 600 sccm, (**b**) 1 bar and 2000 sccm, (**c**) 300 sccm at 0.4 and 1 bar. (**d**) Typical mass spectra time traces, here that applied for Fig. 7a.

By adjusting the flows and measuring conversion and plasma power we were able to obtain our energy efficiency curves. In Fig. 7 we report the conversion and the energy efficiency for pure CO_2_ at different flows and pressures. On the top left pane 7a at the pressure of 400 mbar (supposedly where optimal efficiency had been reported for MW plasmas^2^) and for a larger, 2000 sccm flow at 1 bar on the top right pane, 7b.

In Fig. 7c, bottom left, we compare the data at 300 sccm and different pressure and power. Finally on the bottom right, 7d we report a typical time trace for a pure CO_2_ dissociation experiment, from where the values of conversion and energy efficiency were extracted to build Fig. 7a).

As a general trend we can notice how the efficiency, while generally decreasing with plasma power does increase with the flow when larger powers are applied, indicating the importance of a correct matching, consistent to what is reported in Fig. 1. It is also important to note the fast time response of the system when plasma power is applied and varied, which is promising for the application of fast energy transients. As expected with a constant interaction volume, the larger the flow, the faster the reaction.

Figure 7a shows the CO_2_ conversions at a pressure of 400 mbar and a power between 50 and 850 W. Inlet flows were set at 100, 300, and 600 sccm. At the lowest flow of 100 sccm, a high conversion of 20% was measured at a power of about 50 W, which increased rapidly to level off at a value of 30% at about 500 W and then not varying significantly at higher powers up to 900W. As the fluxes were further increased, the conversion curves reached an increasingly linear trend with a progressive decrease in conversions, e.g., at the maximum flux of 600 sccm, the conversion was only 8% at 105 W, to increase to 25% at the maximum power of 800 W. The plasma efficiency showed an opposite trend to that observed for CO_2_ conversion. That is, at the lowest power, a reasonable efficiency of 8% was obtained with the highest flow of 600 sccm, but it decreased to 5% when the flow was reduced to 100 sccm. As the power increased, the efficiency decreased significantly and still reached a value of 4% at the higher flow rate, while it collapsed to less than 1% at the lower flow rate.

Figure 7b shows the conversion and efficiency when a larger flow of 2000sccm is applied. Here the energy efficiency starts at 12% at 200 W, and decreases to 5% at 1000 W, but conversions are lower with an opposite trend from 4% at low power to 11% at 1000 W.

Figure 7c explores the effect of pressure at the flow of 300 sccm, at 400 mbar (red curves) both conversion and energy efficiency are always higher than at 1 bar. The fact that conversions are higher, despite the longer residence time at higher pressure, can be due to the volume increase of the dissociation reaction, but it also points to of less optimal plasma parameters, as the discharge becomes less diffuse and more filamentary at higher pressure^3,5^.

Figure 7d shows the mass spectra time traces that were applied for building the conversion and energy efficiency curves reported in Fig. 7a. The time response of the system to the different plasma conditions is promising for the valorisation of fast electric energy transients. Due to the lack of any thermal inertia in a cold plasma, the trace risetime of all products is simply linked to the apparatus residence time.

**Figure 8:**
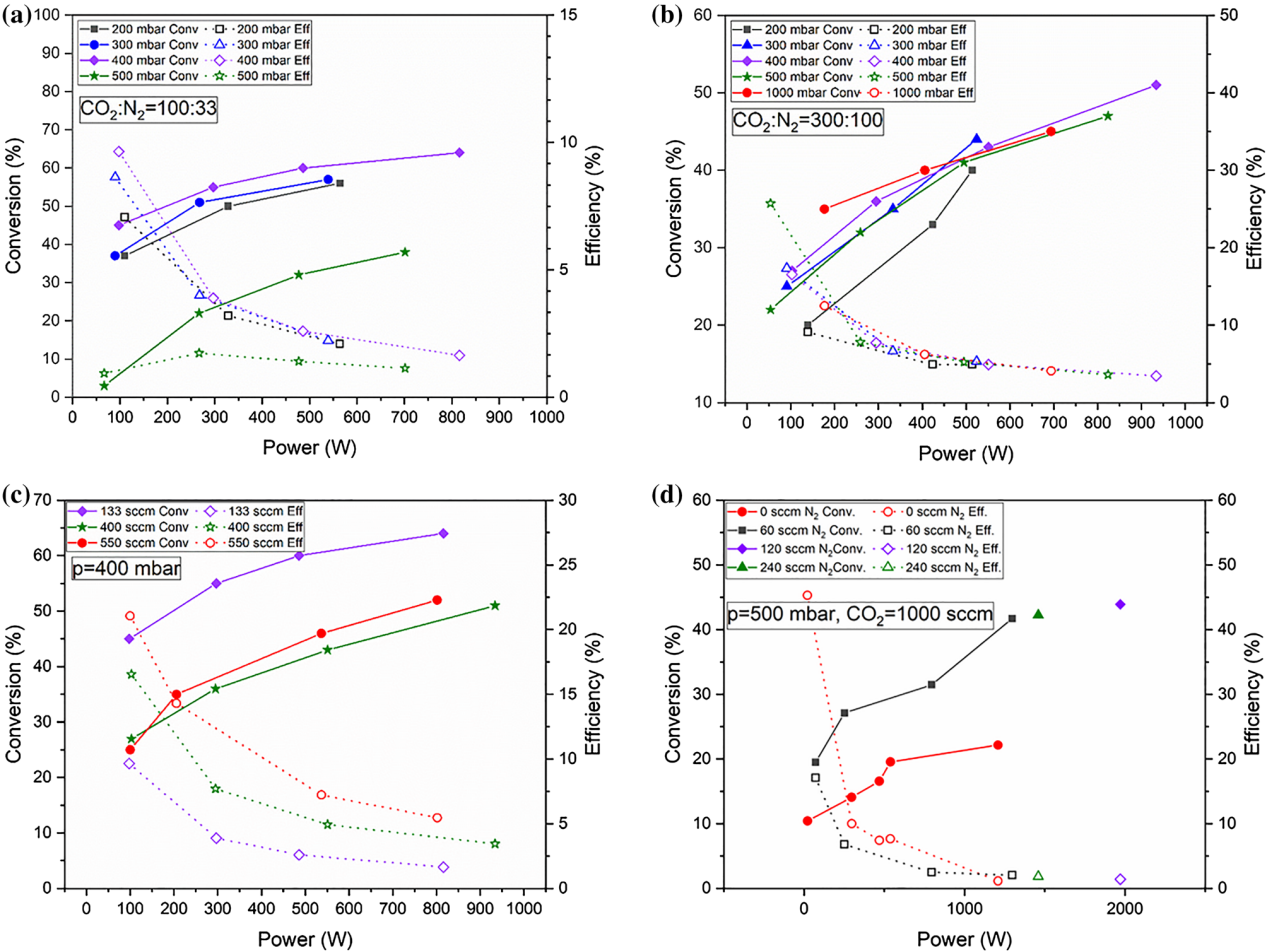
Conversion and efficiencies for CO_2_/N_2_ mixtures, at different pressure, flow, and power: (**a**) effect of pressure at 3/1 ratio and CO_2_ = 100 sccm and, (**b**) 3/1 ratio and CO_2_ = 300 sccm; (**c**) effect for flow at 3/1 ratio and P = 400 mbar; (**d**) effect of different CO_2_/N_2_ ratios at P = 500 mbar and CO_2_ = 1000 sccm.

In Fig. 8 instead conversion rates and energy efficiencies are reported for N_2_/CO_2_ mixtures, at different flows and pressures ranging from 200 mbar to 1 bar. On the top row for a fixed 3:1 ratio: 8a on the left for a smaller flow of 133 sccm and on the right, 8b for 400 sccm. Again, on the bottom left 8c the effect of the total flow for the 3:1 mixture at 400 mbar.

We also studied the effect of varying amounts of N_2_ with respect to a large, pure 1000 sccm CO_2_ flow as reported in Fig. 8d at 500 mbar.

Considering that we increased the power supply voltage at fixed steps while building the power curves we noticed that adding N_2_ may improve the power coupling to the discharge at lower voltages, indeed we could feed up to 2 kW with 1000 sccm CO_2_ and 240 sccm N_2_.

In Fig. 8a, with a flow of 100sccm of CO_2_ and 33 sccm of N_2_, at 400 mbar we reach a conversion above 60%, at 800 W, the energy efficiency starts at 10% at 100 W and bottoms at 2% at 800 W.

Very similar trends are observed for conversion and efficiency at all pressures in Fig. 8b, where the flow is 300sccm of CO_2_ and 100 sccm of N_2_, larger efficiencies and conversions are observed at 400 mbar and 500 mbar with respects to lower and higher pressures. Here, a 26% energy efficiency with a conversion of 22% at is observed at low power at 500 mbar, while still at low power we have 36% conversion and 22% efficiency at 1 bar. The 400-mbar case is detailed in Fig. 8c at different flows. Here higher flows yield higher energy efficiency and lower conversions due to discharge power mismatches to the reactant flow enthalpy. At 400 sccm CO_2_ and 150 sccm N_2_ (total 550 sccm) a good trade-off is observed for conversion and efficiency, respectively 25% and 22.5% at 100 W and 35% and 15% at 230 W.

In Fig. 8d we explore the effect of different addition of N_2_ to a large 1000 sccm flow of CO_2_ at 500 mbar. Here we could couple to the plasma up to 2 kW for a 45% conversion, but a low 3% efficiency. By evaluating the red and black curves, respectively with naught and 60 sccm of N_2_ we notice as N_2_ favors conversion but not efficiency.

The results obtained in this work are clearly consistent to the ones found in the literature, that was recently reviewed by Snoeckx and Bogaerts^34,35^: the general trends above described are confirmed and they are a characteristic of DBD reactors also for higher power resonating plasmas. Consistently most of our results are in the range below 15% efficiency at higher conversion, identified as threshold in the cited article^34,35^. In the present work we achieved a good combination of conversion and efficiency at 36% and 17.3% respectively (red curve in Fig. 8b) at atmospheric pressure, close the best values reported^34,35^ at 42% conversion and 23% efficiency, with a packed bed DBD reactor.

We observed also that the injection of N_2_ in the gas mixtures seems to improve the conversion of the reaction due to the dissociation of CO_2_ upon collision with N_2_ metastable molecules, and this effect is strong enough to compensate for the lower CO_2_ content in the mixture^34,35^. The formation of polluting gases such as N_2_O and NO_x_ formed as reaction by-products in the order of some hundreds of ppm can give rise to an environmental problem. However, the feeding mixtures of a future DBD reactor under industrially relevant conditions should consider the presence of N_2_ since the most common exhausted gases contain some amount of this gas.

**Figure 9:**
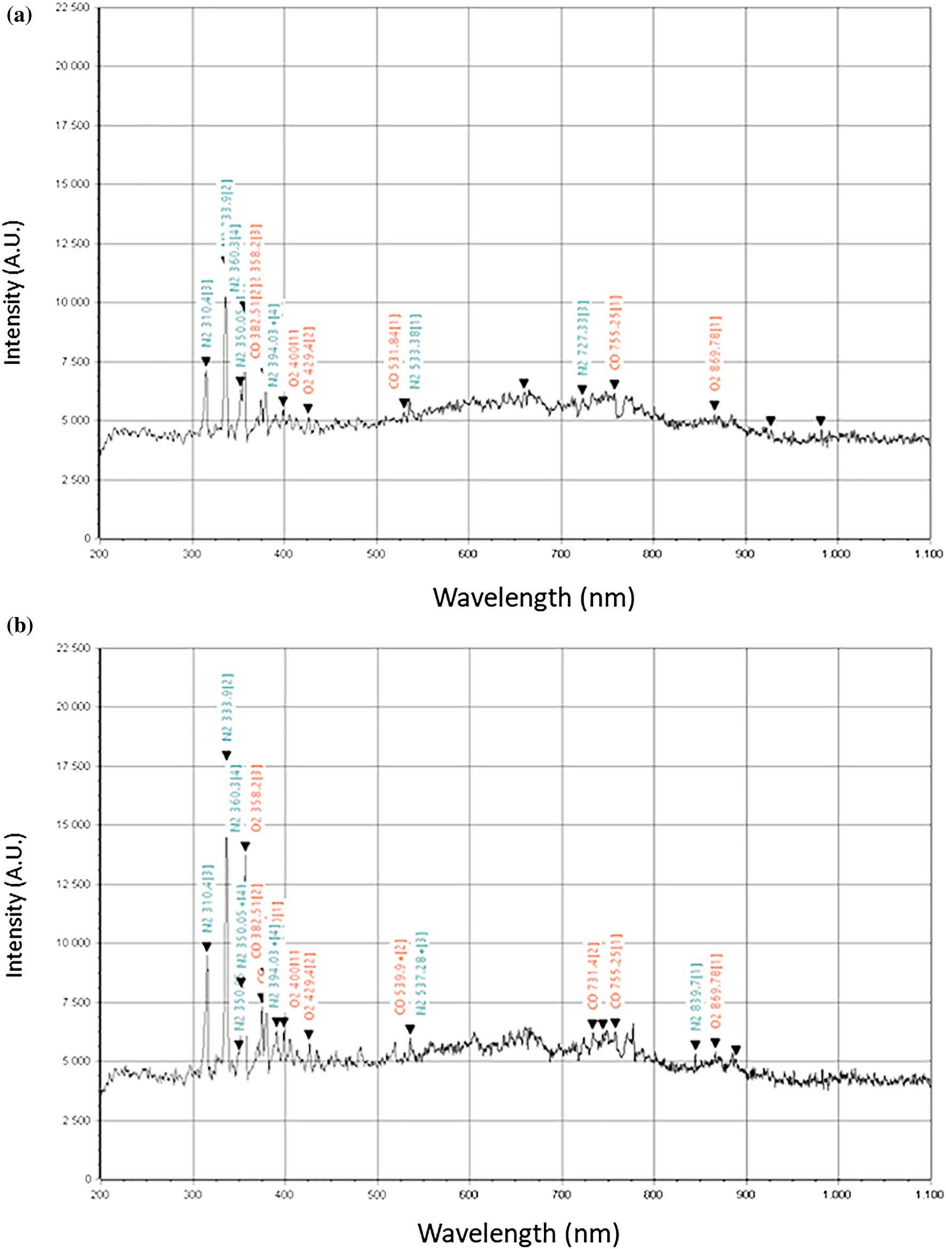
Optical Emission Spectra of discharges in N_2_/CO_2_ = 300/100 at 400 mBar (for green/star-point curve of Fig. 8c): (**a**) 100W Eff = 16%; (**b**) 930W Eff = 3.4%.

As application examples of OES spectroscopy, in Fig. 9 we show the optical emission spectrum of the discharge operating in N_2_/CO_2_ at low power and high efficiency (respectively 100 W and 16% in Fig. 9a) and low efficiency and high power (930 W and 3.5% in Fig. 9b). The spectra were collected while building the green (star points) conversion efficiency curve of Fig. 8c.

We observed that the discharge remains mainly dark except for the intense emission of N_2_ in the UV-blue part of the spectrum. Increasing the power, a more intense optical emission is observed in general and the onset of emission from CO between 700 and 800 nm. If any electronic excitation and optical emission in the eV range represents an energy loss and indicates efficiency dop, we could not attribute to directly to that the large drop of energy efficiency with power. However, it indicates that the electron energy distribution exceeds the sub eV range where CO_2_ dissociation is most efficient^1,2^.

**Figure 10:**
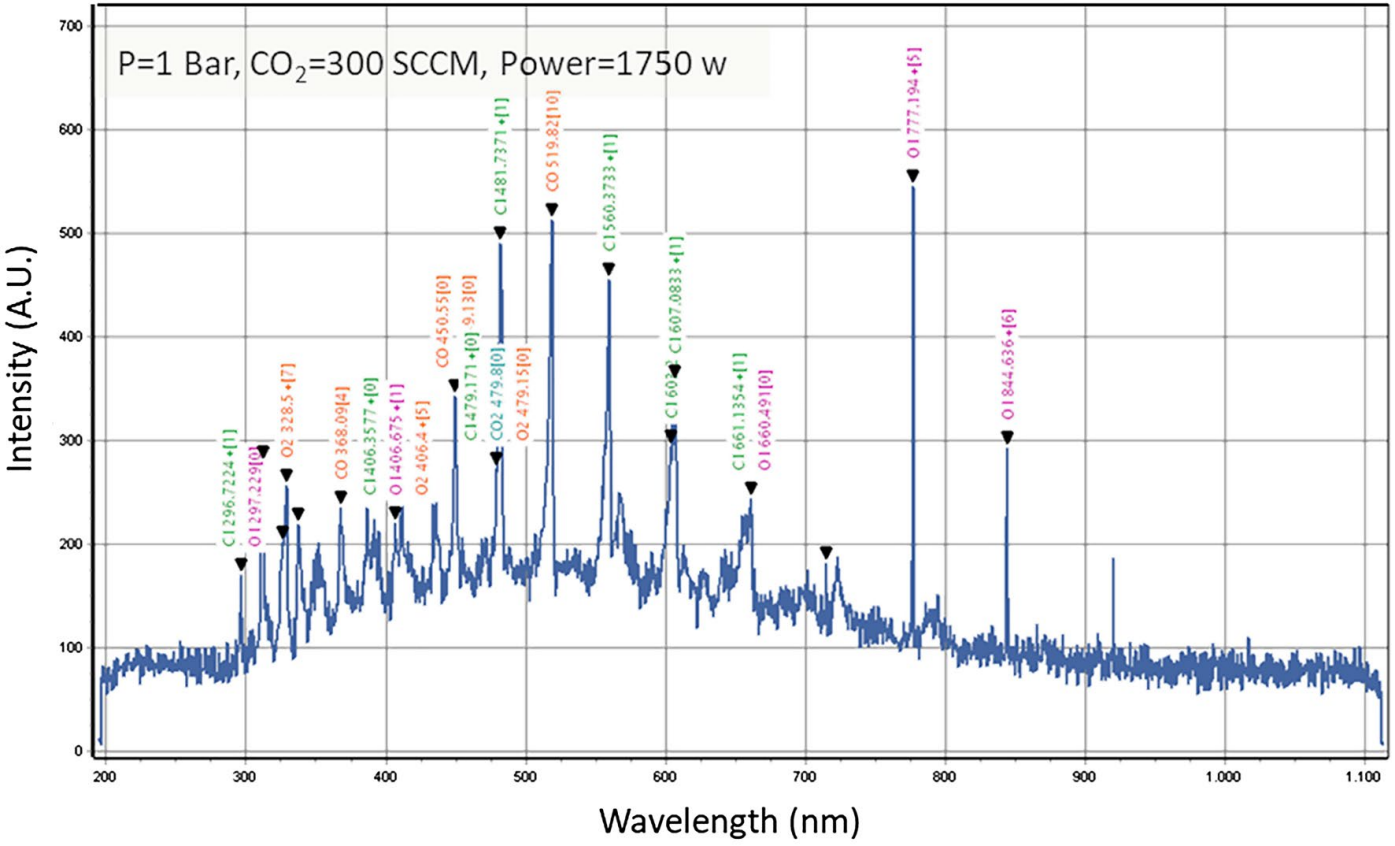
Optical Emission Spectrum of a high-power discharge plasma in pure CO_2_: P = 1 Bar, CO_2_ = 300 sccm, 1750 W. The presence of intense optical emission from CO in the 1 eV range, besides atomic lines indicate excess electronic excitation and molecular cracking, leading to energy inefficient dissociation.

In a pure 300 sccm CO_2_ flow, high power 1750 W discharge at 500 mbar, the optical emission in the UV–visible is substantially increased as seen in Fig. 10, whereas the discharge is mostly dark at lower power. At this power level, which is well in excess to that strictly needed for the complete dissociation of that CO_2_ flow (see Fig. 1) we experienced the presence of strong emission lines from CO and O_2_, but also atomic and ionic lines from C and O.

The ionization from high energy electrons and the emission of light with energy above 1 eV indicates that the discharge is operating in an inefficient regime for the CO_2_ dissociation. Conversely, if the CO_2_ is dissociated by collisions with electrons with 1 eV and less via successive vibrational excitation into self-dissociative states^2^, then the discharge should remain “dark”, mostly free of emission lines in the visible wavelength. Also, no emission lines from C and O atoms, nor from CO, O_2_, C and O ions should be observed, and a sensitive online OES diagnostic can be effective to detect the incipit of different discharge regimes, energetically inefficient for CO_2_ dissociations. Further, by improving light collection OES can be applied to measure gas phase temperature and electron density as a real-time diagnostic for optimizing direct CO_2_ plasma dissociation.

In our water cooled DBD system the OES measurements identify the onset of less efficient discharge regimes for the purpose of pure CO_2_ dissociation into O_2_ and CO. However, it is not still clear if the efficiency drop is caused by the phenomena behind the occurrence of optical emission lines, given that relatively little energy is conveyed to emission relatively the discharge power, also in view that a small electron population (in the high energy tail of the electron energy distribution) can cause that emission.

Aiming at improving the system to increase efficiency we first observe that plasma power should be always matched to the gas flow dissociation enthalpy, and we see that efficiency increases at all power levels in Figs. 7a,c and 8c, so that working efficiently at high power would require flows larger than those explored in the current study.

Among the other possible reasons for the efficiency drop at higher power is the increase in temperature of the electrode surfaces, where the back-reaction (CO and O_2_ in CO_2_) may be favored, which can be limited applying other materials and improving the machine design. A simple calculation that considers the cylindrical heat conduction in the current geometry, that neglects heat conduction losses between electrode and the cooled coaxial support, indicates that our stainless-steel electrode surface reaches about 115 °C with a power of 1 kW and more than 200 °C at 2 kW.

Other important plasma parameters may be offset from optimal values and can be tuned and optimized^36^, the reduced electric field E/N_0_, probably too high^2,28,37^. For a homogeneous, streamer free discharge the reduced electric field could be estimated from the voltage and the current waveform by dividing the measured voltage at the current onset by gap and pressure. The numbers that we obtain are in the range 1–3 × 10^–15^ vcm^2^ that imply a regime of electronic excitation rather than vibrational. For inhomogeneous, streamer dominated discharges the picture is more complex as E/N_0_ is not the same across the discharge. Also and the individual streamer micro-discharge “pulse” duration is probably too short, here preliminary current pulse measurements point to current spikes of 10 ns or less while a longer time is required for the stepwise CO_2_ dissociation^2,38^.

## Conclusions and outlook

We have designed, constructed, and developed a novel dielectric barrier discharge system, based on water cooled electrodes which is capable to explore interesting high power (2 kW) and energy efficiency regimes. Energy efficiencies of CO_2_ dissociation were measured up to and more than 30% at low power, and conversion rates up to 60% when higher power is applied. The key investigation for future development will be the exploration of regimes that allow to simultaneously increase the energy efficiency and the conversion rate for larger CO_2_ flows where high power is applied to the plasma. This can be accomplished by further limiting the back reaction of CO and O_2_ by means of: (i) a better thermal management of the electrodes and surface engineering; (ii) the optimized matching of the gas flow to the discharge volume and power; (iii) inserting a membrane to separate the O_2_ from the plasma. Further, we consider that DBD systems act as a collection of micro-discharges^38^, that collapse in the course of each AC cycle into several streamers in a matter of nanoseconds: the implementation of discharges with different micro-discharge spatial and temporal regimes, which would bring electron density and reduced field (E/N_0_) closer to optimal values for CO_2_ dissociation might greatly benefit the process, with energy efficiencies that may become viable for practical purposes. may greatly benefit the efficiency. Finally, the introduction of different electrode and dielectric materials, using materials with higher heat conductivity and improving the thermal contact with the cooling fluid, and adding catalysts into the discharge region will further increase the application potential of high power, water cooled DBD plasma systems.

## Data Availability

The datasets used and/or analysed during the current study available from the corresponding author on reasonable request.

## References

[1] Belousov IG, Legasov VA, Rusanov VD (1980). A plasmochemical concept for thermochemical hydrogen production. Int. J. Hydrogen Energy.

[2] Fridman A (2008). Plasma Chemistry.

[3] Kogelschatz U, Eliasson B, Egli W, Kogelschatz U, Eliasson B, Principle WEDD (1997). Dielectric-barrier discharges. Principle and applications. J. Phys. Iv.

[4] Kwak HS, Uhm HS, Hong YC, Choi EH (2015). Disintegration of carbon dioxide molecules in a microwave plasma torch. Sci. Rep..

[5] Brandenburg R (2018). Dielectric barrier discharges: Progress on plasma sources and on the understanding of regimes and single filaments. Plasma Sour. Sci. Technol..

[6] Staack D, Farouk B, Gutsol A, Fridman A (2005). Characterization of a Dc atmospheric pressure normal glow discharge. Plasma Sour. Sci. Technol..

[7] Renninger S, Stein J, Lambarth M, Birke KP (2022). An optimized reactor for CO_2_ splitting in DC atmospheric pressure discharge. J. CO2 Util..

[8] Navascués P, Cotrino J, González-Elipe AR, Gómez-Ramírez A (2021). Plasma assisted CO_2_ dissociation in pure and gas mixture streams with a ferroelectric packed-bed reactor in ambient conditions. Chem. Eng. J..

[9] Li S, Ongis M, Manzolini G, Gallucci F (2021). Non-thermal plasma-assisted capture and conversion of CO_2_. Chem. Eng. J..

[10] Centi G, Perathoner S, Papanikolaou G (2021). Plasma assisted CO_2_ splitting to carbon and oxygen: A concept review analysis. J. CO2 Util..

[11] Zhang H, Li L, Li X, Wang W, Yan J, Tu X (2018). Warm plasma activation of CO_2_ in a rotating gliding arc discharge reactor. J. CO2 Util..

[12] Michielsen I, Uytdenhouwen Y, Pype J, Michielsen B, Mertens J, Reniers F, Meynen V, Bogaerts A (2017). CO_2_ dissociation in a packed bed DBD reactor: First steps towards a better understanding of plasma catalysis. Chem. Eng. J..

[13] Ding W, Xia M, Shen C, Wang Y, Zhang Z, Tu X, Liu CJ (2022). Enhanced CO_2_ conversion by frosted dielectric surface with ZrO_2_ coating in a dielectric barrier discharge reactor. J. CO2 Util..

[14] Pietanza LD, Colonna G, Capitelli M (2022). Non-equilibrium plasma kinetics of CO_2_ in glow discharges: A comparison with existing modeling and experimental results. Plasma Sour. Sci. Technol..

[15] Savinov SY, Lee H, Song HK, Na BK (2002). The decomposition of CO_2_ in glow discharge. Korean J. Chem. Eng..

[16] Li L, Zhang H, Li X, Kong X, Xu R, Tay K, Tu X (2019). Plasma-assisted CO_2_ conversion in a gliding arc discharge: Improving performance by optimizing the reactor design. J. CO2 Util..

[17] Nunnally T, Gutsol K, Rabinovich A, Fridman A, Gutsol A, Kemoun A (2011). Dissociation of CO_2_ in a low current gliding arc plasmatron. J. Phys. D Appl. Phys..

[18] Paunska T, Trenchev G, Bogaerts A, Kolev S (2019). A 2D model of a gliding arc discharge for CO_2_ conversion. AIP Conf. Proc..

[19] Zhang H, Li L, Xu R, Huang J, Wang N, Li X, Tu X (2020). Plasma-enhanced catalytic activation of CO_2_ in a modified gliding arc reactor. Waste Dispos. Sustain. Energy.

[20] Wang W, Mei D, Tu X, Bogaerts A (2017). Gliding arc plasma for CO_2_ conversion: Better insights by a combined experimental and modelling approach. Chem. Eng. J..

[21] De Bie C, Van Dijk J, Bogaerts A (2016). CO_2_ hydrogenation in a dielectric barrier discharge plasma revealed. J. Phys. Chem. C.

[22] Wang J, Wang X, AlQahtani MS, Knecht SD, Bilén SG, Chu W, Song C (2022). Synergetic effect of nonthermal plasma and supported cobalt catalyst in plasma-enhanced CO_2_ hydrogenation. Chem. Eng. J..

[23] Zhang F, Zhang X, Song Z, Li X, Zhao X, Sun J, Mao Y, Wang X, Wang W (2023). Promotion of microwave discharge over carbon catalysts for CO_2_ reforming of CH_4_ to syngas. Fuel.

[24] Wang Y, Chen Y, Harding J, He H, Bogaerts A, Tu X (2022). Catalyst-free single-step plasma reforming of CH_4_ and CO_2_ to higher value oxygenates under ambient conditions. Chem. Eng. J..

[25] Mei D, Zhang P, Duan G, Liu S, Zhou Y, Fang Z, Tu X (2022). CH4 reforming with CO_2_ using a nanosecond pulsed dielectric barrier discharge plasma. J. CO2 Util..

[26] Snoeckx R, Wang W, Zhang X, Cha MS, Bogaerts A (2018). Plasma-based multi-reforming for gas-to-liquid: Tuning the plasma chemistry towards methanol. Sci. Rep..

[27] Bongers W, Bouwmeester H, Wolf B, Peeters F, Welzel S, van den Bekerom D, den Harder N, Goede A, Graswinckel M, Groen PW (2017). Plasma-driven dissociation of CO_2_ for fuel synthesis. Plasma Process. Polym..

[28] Legasov VA, Zhivotov VK, Krasheninnikov EG, Krotov MF, Patrushev BI, Rusanov VD, Rykunov GV, Spektor AM, Fridman AA, Sholin GV (1978). Nonequilibrium plasmochemical process of the CO_2_ decomposition in HF and SHF discharges. Dokl. Akad. Nauk SSSR.

[29] Chen G, Buck F, Kistner I, Widenmeyer M, Schiestel T, Schulz A, Walker M, Weidenkaff A (2019). A novel plasma-assisted hollow fiber membrane concept for efficiently separating oxygen from CO in a CO_2_ plasma. Chem. Eng. J..

[30] LTspice Simulator | Analog Devices. https://www.analog.com/en/design-center/design-tools-and-calculators/ltspice-simulator.html.

[31] Beroual A, Khaled U, Coulibaly ML (2018). Experimental investigation of the breakdown voltage of CO_2_, N_2_, and SF_6_ Gases, and CO_2_-SF_6_ and N_2_-SF_6_ mixtures under different voltage waveforms. Energies (Basel).

[32] Pal UN, Kumar M, Tyagi MS, Meena BL, Khatun H, Sharma AK (2010). Discharge analysis and electrical modeling for the development of efficient dielectric barrier discharge. J. Phys. Conf. Ser..

[33] Ashpis DE, Laun MC, Griebeler EL (2017). Progress toward accurate measurement of dielectric barrier discharge plasma actuator power. AIAA J..

[34] Snoeckx R, Bogaerts A (2017). Plasma technology-a novel solution for CO_2_ conversion?. Chem. Soc. Rev..

[35] Bogaerts A, Centi G (2020). Plasma technology for CO_2_ conversion: A personal perspective on prospects and gaps. Front. Energy Res..

[36] Zhu XM, Pu YK (2010). Optical emission spectroscopy in low-temperature plasmas containing argon and nitrogen: Determination of the electron temperature and density by the line-ratio method. J. Phys. D Appl. Phys..

[37] Ozkan A, Dufour T, Silva T, Britun N, Snyders R, Reniers F, Bogaerts A (2016). DBD in burst mode: Solution for more efficient CO_2_ conversion?. Plasma Sour. Sci. Technol..

[38] Ozkan A, Dufour T, Silva T, Britun N, Snyders R, Bogaerts A, Reniers F (2016). The influence of power and frequency on the filamentary behavior of a flowing DBD—Application to the splitting of CO_2_. Plasma Sour. Sci. Technol..

